# Breast Cancer-Derived Microvesicles Are the Source of Functional Metabolic Enzymes as Potential Targets for Cancer Therapy

**DOI:** 10.3390/biomedicines9020107

**Published:** 2021-01-22

**Authors:** Yousef Risha, Vanessa Susevski, Nico Hüttmann, Suttinee Poolsup, Zoran Minic, Maxim V. Berezovski

**Affiliations:** 1 Department of Chemistry and Biomolecular Sciences, University of Ottawa, Ottawa, ON K1N 6N5, Canada; yrish083@uottawa.ca (Y.R.); vsuse027@uottawa.ca (V.S.); nhutt069@uottawa.ca (N.H.); spool093@uottawa.ca (S.P.); 2 John L. Holmes Mass Spectrometry Facility, Faculty of Science, University of Ottawa, Ottawa, ON K1N 6N5, Canada; zminic@uottawa.ca

**Keywords:** breast cancer, proteomics, microvesicles, small extracellular vesicles, transaldolase (TALDO1), ornithine aminotransferase (OAT), bleomycin hydrolase (BLMH)

## Abstract

Membrane-derived extracellular vesicles, referred to as microvesicles (MVs), have been proposed to participate in several cancer diseases. In this study, MV fractions were isolated by differential ultracentrifugation from a metastatic breast cancer (BC) cell line MDA-MB-231 and a non-cancerous breast cell line MCF10A, then analyzed by nano-liquid chromatography coupled to tandem mass spectrometry. A total of 1519 MV proteins were identified from both cell lines. The data obtained were compared to previously analyzed proteins from small extracellular vesicles (sEVs), revealing 1272 proteins present in both MVs and sEVs derived from the MDA-MB-231 cell line. Among the 89 proteins unique to MDA-MB-231 MVs, three enzymes: ornithine aminotransferase (OAT), transaldolase (TALDO1) and bleomycin hydrolase (BLMH) were previously proposed as cancer therapy targets. These proteins were enzymatically validated in cells, sEVs, and MVs derived from both cell lines. The specific activity of OAT and TALDO1 was significantly higher in MDA-MB-231-derived MVs than in MCF10A MVs. BLMH was highly expressed in MDA-MB-231-derived MVs, compared to MCF10A MVs. This study shows that MVs carry functional metabolic enzymes and provides a framework for future studies of their biological role in BC and potential in therapeutic applications.

## 1. Introduction

Breast cancer (BC) continues to be one of the leading causes of death for women worldwide, accounting for 25% of all cancers [1]. Overall mortality rates of BC are decreasing in developed countries due to increased awareness of self-examination procedures, improvement in early detection methods, and treatment advances [2,3]. However, the mortality rates of metastatic BC subtypes remain alarming, with an estimated five-year survival rate of 36% [4].

The development of metastatic BC is characterized by certain hallmarks—modulation of microenvironment, angiogenesis, sustained growth and tissue invasion—that rely on cell–cell signaling and communication [5,6]. Extracellular vesicles (EVs) have emerged as a novel mechanism of intercellular communication that does not require direct cellular contact [7]. Encapsulated by a phospholipid bilayer, these vesicles induce biologically relevant changes in recipient cells by transferring bioactive content (nucleic acids, proteins, and lipids) from donor to nearby or distant cells [8,9,10]. EVs may be classified based on their morphology, cellular origin, or biogenesis pathways. Small EVs (sEVs), including exosomes (Exo), range in size from 30 to 150 nm [11,12]. On the other hand, microvesicles (MVs) are formed by direct outward budding of the cell membrane and are 50 nm to >1000 nm in diameter [13]. In this study, vesicles of a size between 100 and 500 nm are referred to as MVs.

Small EVs play an important role in cancer progression by supporting tumor metastasis, drug resistance, and immune system evasion [14,15,16,17]. Similarly, MVs contribute to cancer progression; however, their precise role is less understood [18,19,20]. Pancreatic cancer patients’ serum MVs enhanced the proliferation and migration of PANC-1 cells in-vitro [18], while squamous cell carcinoma-derived MVs enhance endothelial cell angiogenesis [19]. Moreover, evidence suggests that MVs may assist in the early detection of cancer. The expression levels of mucin 1 (MUC1), epidermal growth factor receptor (EGFR), epithelial cell adhesion molecule (EpCAM), and extracellular matrix metalloprotease inducer (EMMPRIN) in tumor-derived MVs were used to distinguish cancer patients from healthy controls regardless of tumor type [20].

MVs also play important roles in BC through the transfer of oncogenic molecules. For example, miRNA221 was found within triple-negative breast cancer (TNBC) MVs [21]. This microRNA promotes epithelial to mesenchymal transition of nonmetastatic BC cells, such as MCF7, by reducing E-cadherin expression [22]. BC tumor-derived MVs, but not MVs derived from benign mammary cells, enhance cancer cell invasion via the activation of the p38/MAPK pathway [23]. Finally, MVs secreted from multi-drug resistant BC cells can aid in immune evasion by transforming macrophages to an incapacitated state [24].

The global study of proteins using modern proteomic methods has provided a deeper understanding of many diseases [25,26,27]. EV proteomics can elucidate the biological role of EVs in BC progression and therapy. Proteomics revealed a panel of nine MV proteins used to classify individuals with cancer at 70% sensitivity and 76% specificity [28]. Furthermore, MV proteomic profiling elucidated possible drug resistance mechanisms in BC through the transfer and stabilization of P-glycoprotein [29].

In this study, the proteomes of the metastatic BC cell line MDA-MB-231-derived MVs were compared to the non-cancerous cell line MCF10A-derived MVs. Differences were identified in the MV proteins when compared to the previously profiled sEV proteome derived from the same metastatic BC cell line MDA-MB-231 [30]. There are significant differences in the proteomes of BC-derived exosomes and MVs. Three enzymes unique to MDA-MB-231 were validated in cells, sEVs, and MVs.

## 2. Experimental Section

### 2.1. Cell Culturing

Cell lines used for this study were MDA-MB-231 female epithelial breast cancer cells (ATCC HTB-26) and MCF10A non-tumorigenic epithelial breast tissue cells (ATCC CRL-10317). MDA-MB-231 cells were maintained in high-glucose Dulbecco’s modified Eagle’s medium (11965126, Life Technologies Inc., Burlington, ON, Canada) and supplemented with 10% fetal bovine serum (MT35015CV, Corning, Manassas, VA, USA). MCF10A cells were grown in DMEM/Nutrient Mixture F-12 (11330032, Life Technologies Inc., Burlington, ON, Canada) supplemented with 5% horse serum (HS), 20 ng/mL epidermal growth factor (EGF), 0.5 mg/mL hydrocortisone, 100 ng/mL cholera toxin, and 10 µg/mL insulin. All media used had 100 units/mL penicillin and 0.1 mg/mL streptomycin (LS15070063, Fisher Scientific, Nepean, ON, Canada) added to them. Cells were incubated at 37 °C with 5% CO_2_.

### 2.2. Vesicle Collection

Cells were grown in complete media. When cells reached 40–50% confluency, they were rinsed with Dulbecco modified phosphate buffered saline (PBS) and incubated with vesicle depleted media for 48 h. Vesicle depleted media was prepared by ultracentrifugation of FBS or HS at 100,000× *g* for 20 h. The vesicle-enriched media was then collected and processed for the isolation of MVs.

### 2.3. Differential Ultracentrifugation (UC)

MVs were isolated using differential ultracentrifugation (Figure S1). Then, 100 mL of cell culture supernatant were collected and immediately centrifuged at 300× *g* for 10 min. This was followed by a 3000× *g* spin on a Sigma13190 rotor (MBI) for 10 min, then a 15,000× *g* centrifugation for 35 min using an SW28 swinging bucket rotor (Beckman Coulter). The MV-containing pellet was collected after the 16,000× *g* spin in PBS. The collected MVs were washed by centrifuging at 15,000× *g* for 1 h using an SW55Ti swinging bucket rotor (Beckman Coulter). The sedimented MVs were finally resuspended in PBS.

### 2.4. Quantification of Microvesicles by Nanoparticle Tracking Analysis (NTA)

The ZetaView nanoparticle tracking microscope PMX-110 (Particle Metrix) was used for determining the concentration and size distribution of EVs at 85 and 70 camera shutter speeds. Maximum and minimum sizes were set to 1000 and 20 nm, respectively. Minimum brightness was adjusted to 30, while frames per second were set to 15. Polystyrene beads 350 nm in diameter (CPC400E, IZON, Medford, MA, USA) were used to focus the instrument and ensure proper quantification readings.

### 2.5. Sample Preparation & Nano-LC-MS/MS

Samples were lysed, reduced, alkylated, digested, and analyzed by an Orbitrap Fusion mass spectrometer (Thermo Fisher Scientific, Mississauga, ON, Canada) coupled to an UltiMate 3000 nanoRSLC (Thermo Fisher Scientific, Mississauga, ON, Canada) as described previously [29].

### 2.6. MS Data Processing

MS raw files were analyzed using MaxQuant version 1.6.12.0 [31]. Peptides were searched against the human UniProt FASTA database using the Andromeda search engine [32], integrated into MaxQuant. Default parameters were used if not described otherwise. Oxidation of methionine and N-terminal acetylation were set as variable modification and carbamidomethylation of cysteine as a fixed modification. Trypsin and LysC, C-terminal cleavage at lysine and arginine, were set as digestion enzymes to generate peptides of at least 7 amino acids with a maximum of 2 missed cleavages. Identified peptides had an initial precursor mass deviation of up to 7 ppm and a fragment mass deviation of 20 ppm. The false discovery rate (FDR) for peptides and proteins of 1% was determined using a reverse sequence database. Label-free protein quantification (LFQ) values were obtained through the MaxLFQ algorithm [31], considering only unique peptides. A contaminants database provided by MaxQuant was used. All proteins matching the reverse database or labeled as contaminants were filtered out. 

### 2.7. Bioinformatics Analysis

The Maxquant *proteinGroups* output file was used for all further analysis. Protein groups were simplified by only considering the first protein. Gene Ontology (GO) functional annotations and Kegg pathway analysis were obtained using the David 6.8 database [33]. Further analysis and visualization were done in the R programming language using the packages *ggplot2* and *VennDiagram* [34,35]. DisGeNET curated database was used to retrieve protein-disease associations [36]. All EV fractions were analyzed by three independent biological replicates and three technical replicates at the nLC-MS/MS level. Protein identification for comparison by Venn diagrams required identification in one technical and at least two biological replicates. Welsh’s *t*-test was used to assess significance between LFQ values between sample groups. Only proteins with complete LFQ observations were used to compare the difference in abundance, which is shown in the volcano plot. Raw intensity values were used to estimate the abundance rank of proteins unique to MDA-MB-231 MVs.

### 2.8. Data Availability

All MS raw data were submitted to the PRIDE repository (Accession:PXD023505) at the European Bioinformatics Institute.

### 2.9. Flow Cytometry

Vesicle-enriched media was cleared from cell debris and large vesicles by centrifugation at 300× *g* for 10 min, 3000× *g* for 20 min, followed by 15,000× *g* for 20 min. The cell culture media was diluted with PBS in a 1:4 ratio to avoid the swarm effect. Copine-8 (Cat No. ITT1060, G-Biosciences, St. Louis, MO, USA) conjugated with Alexa 647, and Anti-Calreticulin antibody (Cat No. EPR3924, Abcam, Boston, MA, USA) conjugated to PE, were titrated to find the optimal antibody concentration 1/100,000 and 1/10,000, respectively. The diluted supernatant was incubated with the antibodies for 1 h at 140 rpm at room temperature. The complete growth media was filtered using a Steri-flip vacuum system (Cat No. C238, Sigma-Aldrich, Oakville, ON, Canada) and stained by the antibodies as the baseline control for fluorescence. The samples were analyzed using the Beckman Coulter MoFlo Astrios-EQ Cell Sorter obtaining 100,000 events, and the data were processed using Kaluza software (version 2.1, Beckman Coulter, Mississauga, ON, Canada). The gating procedure included doublet discrimination based on forward scatter, followed by reduction of background signal from antibody in vesicle depleted media. For confirmation of vesicle analysis, samples were incubated in 0.1% Triton X-100 for 20 min at room temperature prior to flow cytometry (FC).

### 2.10. Ornithine Aminotransferase Activity Assay

The fractions of cells and EVs were treated with Triton X-100 (0.4% *v*/*v*), centrifuged at 2000× *g* for 1 min. The supernatants were then used for protein estimation using the Bradford assay [37]. The activity of ornithine aminotransferase was determined using a protocol similar to that of Kumar et al. [38]. The standard conditions were 35 mM l-ornithine (O2376, Sigma-Aldrich, Oakville, ON, Canada), 5 mM alpha-ketoglutarate (75890, Sigma-Aldrich, Oakville, ON, Canada), and cofactor 0.05 mM pyridoxal phosphate (82870, Sigma-Aldrich, Oakville, ON, Canada) in 50 mM Tris-HCl (pH 8.0) of a total volume of 1 mL. The reaction mixture was incubated at 37 °C for 30 min. The reaction was stopped by adding 0.25 mL of 3.6 N perchloric acid (311421, Sigma-Aldrich, Oakville, ON, Canada) and 0.25 mL of 2% ninhydrin (151173, Sigma-Aldrich, Oakville, ON, Canada) in water and proceeded as described previously [38]. Controls were stopped at time 0.

### 2.11. Transaldolase Activity Assay

The fractions of cells and EVs were treated with n-Dodecyl β-d-maltoside (DDM) (0.1% *v*/*v*), centrifuged at 2000× *g* for 1 min. Transaldolase enzyme activity determination was carried out using a protocol similar to those of Tsolas and Joris [39] in the presence of 4 mM d-fructose 6-phosphate (F3627, Sigma), 0.2 mM erythrose 4-phosphate (E0377, Sigma), 0.1 mM NADH (10107735001, Sigma-Aldrich, Oakville, ON, Canada), 1 mM MgCl_2_, and 5 units of α-glycerophosphate dehydrogenase/triosephosphate isomerase (G1881, Sigma-Aldrich, Oakville, ON, Canada) in 1 mL PBS (pH 7.4) at room temperature. The decrease in the characteristic absorbance of NADH at 340 nm (ε = 6290 M^−1^ cm^−1^) was monitored for 30 min.

### 2.12. Measurement of Human Bleomycin Hydrolase

The amount of BLMH in various samples was determined according to the protocol of the Thermo Scientific Human BLMH ELISA (enzyme-linked immunosorbent assay) kit (EH46RB, Life Technologies Inc., Burlington, ON, Canada). Different cell and vesicle samples were diluted using assay diluent and processed as described in the protocol. The amount of BLMH was calculated as ng of BLMH per 100 µg of protein in samples.

## 3. Results

### 3.1. Characterization of MVs

**Figure 1:**
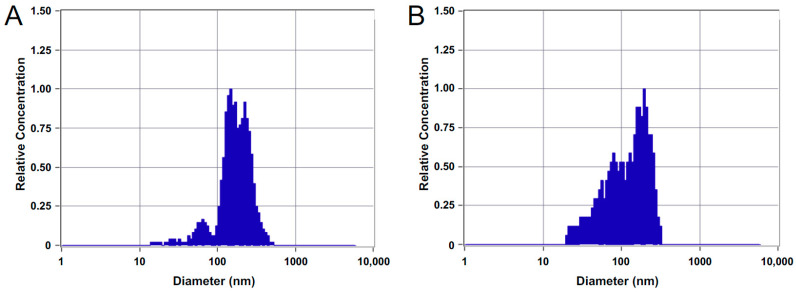
Nanoparticle tracking analysis (NTA) characterization showing size distributions of isolated microvesicles (MVs) from (**A**) MCF10A and (**B**) MDA-MB-231 cell lines.

In our previous study, we profiled MCF10A and MDA-MB-231-derived sEV proteomes isolated by differential ultracentrifugation [30]. Here, we focused on MVs derived from MCF10A and MDA-MB-231 cell lines, which were isolated by collecting the 15,000 g pellet (Figure S1). MCF10A MVs ranged from 75 to 475 nm in diameter with a mode at 169 nm and had a concentration of 1.6 × 10^8^ particles/mL. MDA-MB-231 MVs ranged in diameter of 75 to 410 nm with a mode at 187 nm and a higher number of particles below 100 nm at a concentration of 1.2 × 10^8^ particles/mL (Figure 1).

### 3.2. Proteomics of Cell Line-Derived MVs

MVs derived from MCF10A and MDA-MB-231 cell lines were analyzed, and 1427 and 547 proteins were identified in the MDA-MB-231 and MCF10A-derived MVs, respectively (Table S1, Figure S2). In total, 455 proteins were common to both MDA-MB-231 and MCF10A MVs. Common proteins were excluded from the GO annotations (biological process and cellular localization) enrichment analysis. The “extracellular exosome” cellular localization label was highly enriched in both proteomes (Figure S3A,B). Enrichment of mitochondrial proteins was found in the MCF10A MV proteome. This was evident by cellular localization terms such as “mitochondrion”, “mitochondrial inner membrane” and “mitochondrial respiratory chain complex III.” Biological process terms identified from the MCF10A MV proteome supported the enrichment of mitochondrial proteins with terms that included “mitochondrial electron transport, ubiquitin to cytochrome c”, “aerobic respiration” and “hydrogen ion transport”. The MDA-MB-231 MV proteins mainly originated from the cytosol, membrane, and ribosome according to their cellular localization terms. The GO cellular localization term “mitochondrion” was also identified with a *p*-value of 1.3 × 10^−2^. Biological process terms related to the MDA-MB-231 MV proteome included terms related to translation: “translation initiation” and “SRP-dependent co-translational protein targeting to membrane”. Energy-related biological process terms included “ATP hydrolysis coupled proton transport” with a *p*-value of 7.8 × 10^−3^ and “positive regulation of ATPase activity” with a *p*-value of 2.7 × 10^−2^. The Wnt and NF-kappaB pathways were also represented with terms such as “Wnt signaling pathway, planar cell polarity pathway” and “NIK/NF-kappaB signaling” (Figure S3C,D).

The unique MDA-MB-231 MV proteins were searched against the DisGeNET human diseases database. Out of 972 MDA-MB-231 MV proteins, 112 were cancer-related while 32 were specifically associated with BC (Table S2). In the MDA-MB-231 MV proteome, 23 Wnt signaling pathway proteins were identified based on their GO biological process, five of which—Rho associated coiled-coil containing protein kinase 2 (ROCK2), casein kinase 2 alpha 1 (CSNK2A1), casein, kinase 2 alpha 2 (CSNK2A2), catenin beta 1 (CTNNB1), and glycogen synthase kinase 3 beta (GSK3B)—have a known cancer association. The DisGeNET identified cancer proteins also included five eukaryotic translation initiation factors (EIFs), three of which were BC associated (Eukaryotic translation initiation factor 1A, X-chromosomal (EIF1AX), Eukaryotic initiation factor 4A-II (EIF4A2), and Eukaryotic translation initiation factor 5A-1 (EIF5A)). Moreover, eleven kinases were cancer-associated, some of which include FYN proto-oncogene tyrosine kinase (FYN), aurora kinase B (AURKB), and integrin-linked kinase (ILK).

### 3.3. Comparative Proteomic Analysis of sEVs and MVs

**Figure 2:**
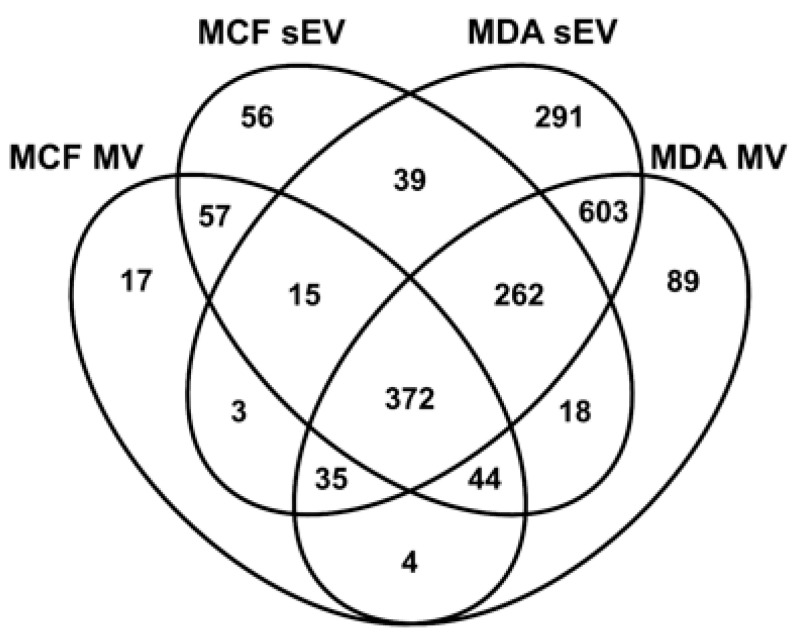
The Venn diagram shows the number of overlapping proteins between the different extracellular vesicle (EV) fractions.

In this study, sEV and MV proteins from MDA-MB-231 and MCF10A cell lines were compared using data from a previous study [30]. In total, 372 proteins were common to all EV fractions (Figure 2). There is also a notable overlap of 1272 proteins between MV and sEVs derived from the MDA-MB-231 cell line. Finally, 89 proteins were unique to MDA-MB-231-derived MVs (Table S3).

**Figure 3:**
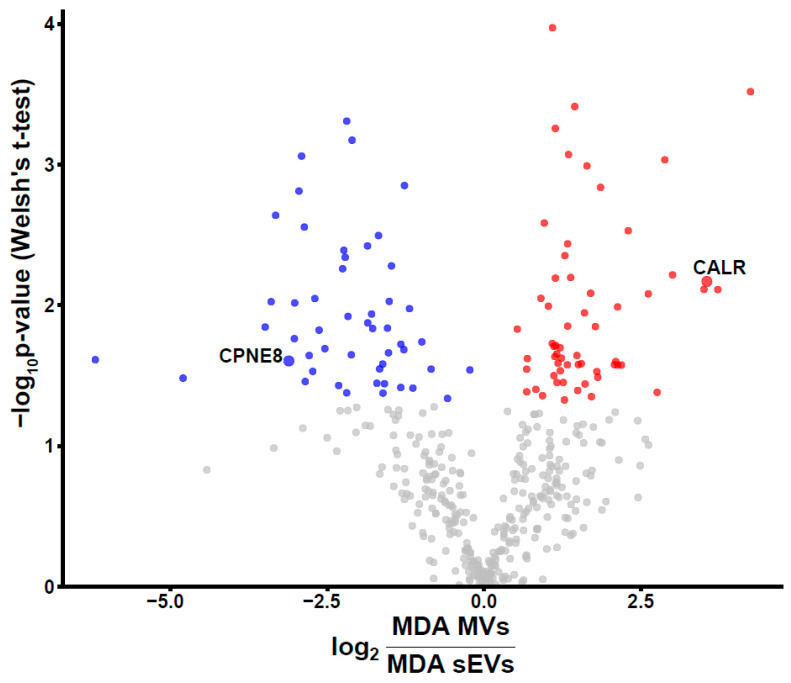
The fold changes of the abundance of proteins common to small different extracellular vesicles (sEVs) and MVs derived from the MDA-MB-231 cell line are depicted in the volcano plot. Proteins with a *p*-value below 0.05 were colored according to higher abundance in sEV (blue) and MVs (red) fractions. *p*-Values were calculated using Welsh’s *t*-test.

In total, 603 proteins were only identified in sEVs and MVs derived from the MDA-MB-231 cell line. To shed light on the quantitative difference of these proteins, fold change and *p*-value were calculated for 420 proteins with complete observations (Figure 3). In total, 50 and 59 proteins were significantly more abundant (*p* < 0.05) in sEVs and MVs, respectively. Copine-8 (CPNE8) and calreticulin (CALR) were selected based on differential expression between MDA-MB-231 MVs and sEVs for flow cytometry (FC) analysis.

**Figure 4:**
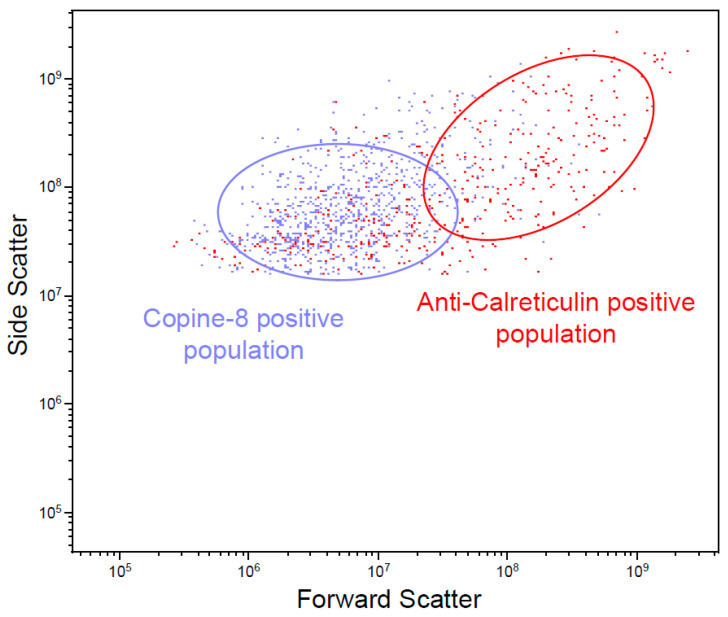
Flow cytometry analysis of EV-containing cell culture supernatant from MDA-MB-231 cells. Ellipses indicate relative scattering difference between CPNE8- and CALR-positive vesicles.

Vesicle-containing cell supernatant was depleted of cells, debris, and larger vesicles before staining with antibodies. Two populations with relative scattering differences confirmed the presence of CPNE8 and CALR on MDA-MB-231-derived EVs (Figure 4). The positive populations are no longer present after treatment of the supernatant with Triton X-100, hence confirming the vesicle nature of identified populations (Figure S4).

### 3.4. Unique BC MV Proteins

**Figure 5:**
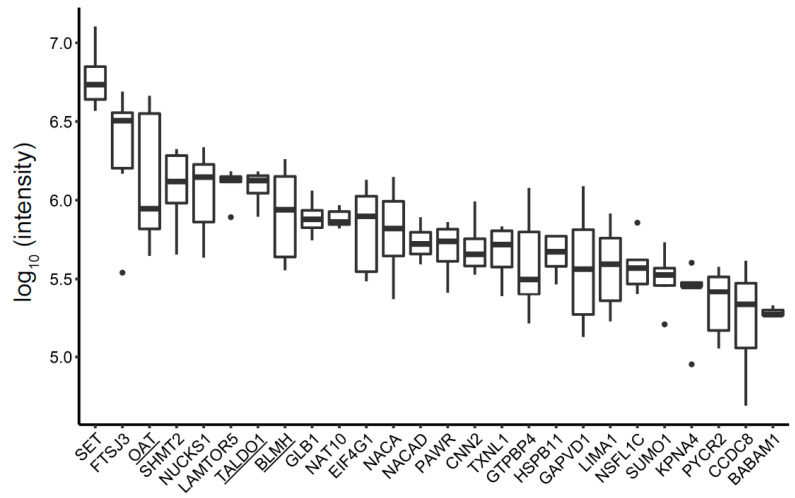
Unique MDA-MB-231-derived MV proteins, ranked according to their intensity, are presented in the boxplot. Underlined proteins were used for validation.

The MDA-MB-231 MV-derived proteome included 89 proteins not found in any of the other protein fractions profiled in this study (Figure 2, Table S3). Three enzymes were significantly abundant in the MDA-MB-231 cell line: aminotransferase enzyme (OAT), transaldolase (TALDO1), and bleomycin hydrolase (BLMH) (Figure 5). These enzymes were further investigated.

### 3.5. Analysis of Ornithine Aminotransferase and Transaldolase in Cells and Their EVs

**Figure 6:**
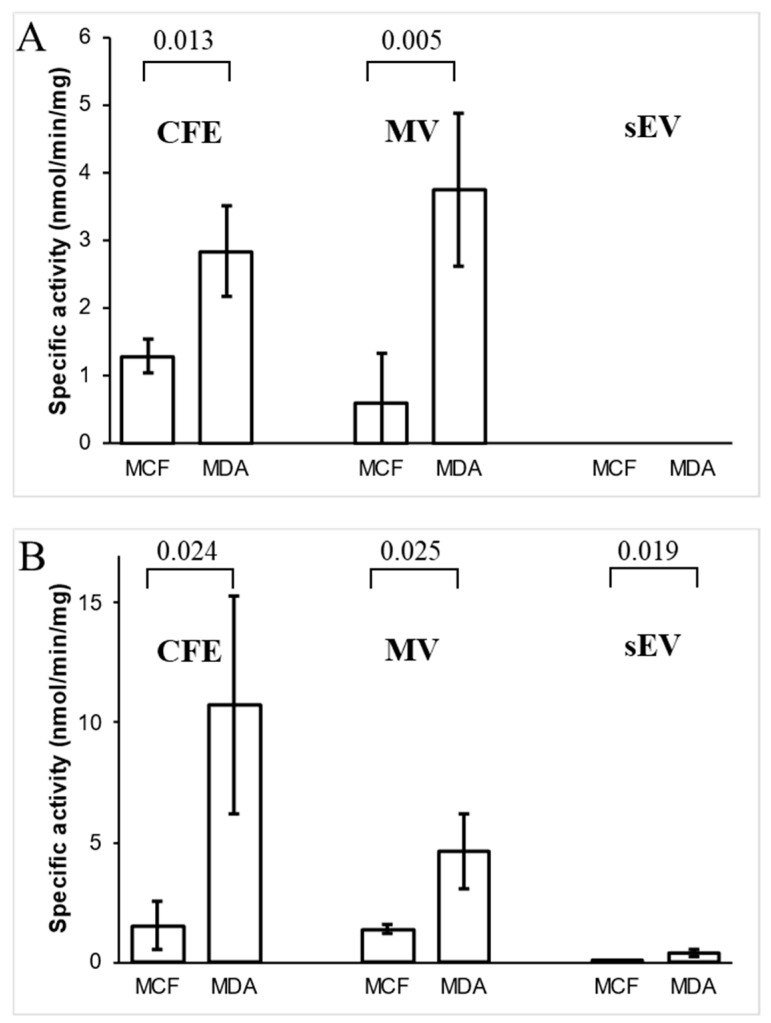
Specific enzymatic activity of (**A**) ornithine aminotransferase (OAT) and (**B**) transaldolase (TALDO1) in cell-free extract (CFE) and their corresponding MV and sEV fractions. The bar graph represents mean values, while error bars indicate the standard deviation (SD) of four replicates, and *p*-values are indicated on the top.

The activity of two enzymes, OAT and TALDO1, only found in the MDA-MB-231-derived MV proteome, were examined. The time course of enzymes for protein extract from MDA-MB-231 cell-free extract (CFE) is presented in Figure S5. OAT activity was detected in both MDA-MB-231 and MCF10A MV fractions, while the sEV fractions from both cell lines did not exhibit any activity for this enzyme (Figure 6A). OAT activity in MDA-MB-231 CFE and MVs was significantly higher when compared to MCF10A CFE and MVs, respectively (Figure 6B). On the other hand, TALDO1 activity was detected in all extracted protein fractions from both cell lines. In contrast to MVs, sEV protein fractions exhibited low TALDO1 activity. Its activity in MDA-MB-231 CFE and MVs was significantly greater than MCF10A’s CFE and MV fractions. These results agree with the proteomics data showing the presence of the enzymes in MDA-MB-231 derived MVs.

### 3.6. Concentrations of Bleomycin Hydrolase in Cells and Their EVs

**Figure 7:**
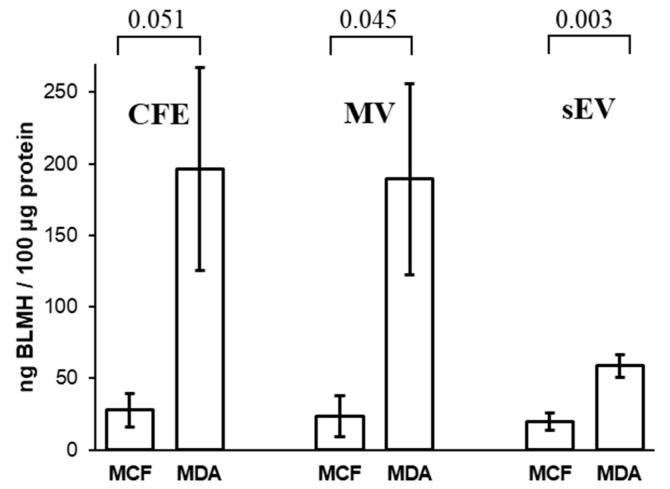
Comparison of amounts of bleomycin hydrolase (BLMH) in MCF10A and MDA-MB-231 CFE and their corresponding MVs and sEVs by ELISA. The bar graph represents mean values, while error bars indicate the standard deviation of three replicates, and *p*-values are indicated on the top.

The results show that the amount of BLMH in MDA-MB-231 CFE is higher in comparison to MCF10A CFE (Figure 7). BLMH was higher expressed in MDA-MB-231 MVs than MCF10A MVs. Relatively low BLMH expression was detected in both MCF10A and MDA-MB-231 sEVs when compared to their corresponding MV fractions. These assessments of BLMH levels by ELISA in MV fractions are in accordance with the proteomics data.

## 4. Discussion

Here we present proteomics analysis of breast cell line-derived EVs to shed light on the significance of MV proteins in cancer. We also utilized proteomics data from previously analyzed MDA-MB-231 and MCF10A cell line-derived sEVs [30].

NTA was used to characterize MVs from the MDA-MB-231 and MCF10A cell lines based on approximate diameters. Consistent with previous studies, the size of isolated MVs, from the 15,000 g pellet, ranged from 75–495 nm (Figure 1A) [40,41,42,43]. Vesicles collected from the 15,000 g pellet had a larger diameter—with the majority of vesicles found at 187 nm—than the previously analyzed sEVs, collected from the 100,000 g pellet, which had the highest concentration of particles at a diameter of 105 nm [30].

Proteomics analysis of MDA-MB-231 and MCF10A-derived MVs revealed the presence of 455 overlapping proteins (Figure S2). Enriched GO biological process terms for MV proteins derived from MDA-MB-231 and MCF10A included terms mainly related to mitochondria. Furthermore, 17 proteins of the 89 proteins unique to MDA-MB-231 MVs (Figure 2) originated from mitochondria according to their cellular localization GO terms (Table S3). Previous reports have shown the presence of mitochondrial proteins and mitochondrial fragments in MVs [43,44]. The biological role of MVs carrying mitochondrial protein is the subject of the current investigation. Reports have shown the ability of these MVs to modulate inflammation. MVs derived from lipopolysaccharide (LPS)-activated monocytes induced a pro-inflammatory response in recipient endothelial cells evident by the expression of type 1 IFN (interferon) and TNF (tumor necrosis factor) genes [45]. Other studies have highlighted the importance of mitochondrial transfer by MVs in improving the metabolism of receiving cells. MVs from bone marrow-derived stromal cells increase ATP concentrations of recipient alveoli in an acute lung injury mouse model [46]. Furthermore, mesenchymal stem cells transfer mitochondria to macrophages through MVs, which increased macrophage phagocytosis and improved metabolism [47].

Previous studies have demonstrated not only the overlap in size between MVs and sEVs but also the presence of common proteins [43,48]. We found 1272 proteins shared between MDA-MB-231-derived sEVs and MVs, from which 603 proteins were unique to the MDA-MB-231 cell line (Figure 2). Out of the 603 shared proteins, we confirmed the higher abundance of Copine 8 (CPNE8) and calreticulin (CALR) in sEVs and MVs, respectively.

Copines are a family of Ca^2+^-dependent, phospholipid-binding proteins found in both plants and animals [49]. CPNE8 was found to be highly expressed in prostate, heart, and brain tissues [50]. It is hypothesized to be a negative regulator of cell proliferation in aggressive acute myelogenous leukemia [51]. Other members of the copine protein family—copine I, III, and V—have been shown to promote several cancers’ migration and growth potentials [52,53,54,55,56].

On the other hand, CALR is expressed in the endoplasmic reticulum, cell membrane, cytoplasm, and nucleus [57]. It participates in several biological processes such as cell adhesion, transcription, and gene expression regulation [58]. In cancer, CALR is proposed as a prognostic marker in gastric cancer and esophageal squamous cell carcinoma [59,60]. Furthermore, its expression levels were found upregulated in oral, vaginal, and ductal breast carcinomas [61,62,63]

Proteomic analysis identified enzymes OAT, TALDO1, and BLMH were only in MVs from metastatic MDA-MB-231 cell line. The specific activity of OAT and TALDO1 was higher in MV fractions of MDA-MB-231 in comparison to the non-cancerous MCF10A cell line-derived MVs. These findings might suggest that these enzymes might play a role in BC.

The OAT enzyme is a mitochondrial protein found in almost all eukaryotic cells. This enzyme converts amino acid L-ornithine and α-ketoglutarate to l-glutamate-5-semialdehyde and L-glutamate. The substrate of OAT, ornithine, is a key substrate for the synthesis of proline, polyamines, and citrulline. Ornithine also plays an important role in the regulation of several metabolic processes leading to diseases like hyperornithinemia, hyperammonemia, gyrate atrophy, and cancer in humans [64]. High levels of ornithine have been reported to be a potential protective factor for BC [65]. Similarly, as we demonstrated for metastatic MDA-MB-231 cell line, OAT is overexpressed in hepatocellular carcinoma, and the inhibition of this enzyme has been suggested to be an effective therapy in mice [66,67]. Moreover, proteomics analysis using two-dimensional differential gel electrophoresis (2D-DIGE) and matrix-assisted laser desorption/ionization-time-of-flight-mass spectrometry (MALDI-TOF-MS) of canine mammary tumors, proposed as a model for human breast cancer, identified OAT as one of several upregulated proteins in metastatic carcinomas [68]. In addition, OAT was found upregulated, 1.3-fold, in small cell lung cancer cell line (NCI-H446) compared to the non-cancerous human bronchial epithelial cell line (16-HBE) [69]. This enzyme’s expression has been correlated to the pathological grade and clinical tumor metastasis stage in lung cancer patients [69,70]. Furthermore, OAT knockout nude mice exhibited significantly suppressed growth and metastasis of lung cancer xenografts [70]. Metastatic MDA-MB-231 cell line-derived MVs exhibiting higher enzymatic activity of OAT than MVs of the non-cancerous MCF10A cell line suggest that cancer-derived MVs may contribute to the overall metabolic status and consequently increase vulnerability in BC.

TALDO1 is an enzyme of the pentose phosphate pathway (PPP) and has been linked to oxidative stress, inflammation, and carcinogenesis [71]. This enzyme transfers three-carbon groups from sedoheptulose-7-phosphate (S7P) to glyceraldehyde-3-phosphate (G3P) to generate erythrose-4-phosphate (E4P) and fructose-6-phosphate (F6P). In fast-dividing cancer cells, this enzyme mediates the PPP that plays a pivotal role in helping glycolytic cancer cells meet their anabolic demands and combat oxidative stress [72]. The differential proteomics of urinary EV proteins showed TALDO1 to be associated with bladder cancer [73]. In hepatomas, TALDO1 activity was increased by 1.5–3.4 times compared to normal liver tissue regardless of tumor stage [74]. TALDO1 is indicative of hepatocellular carcinoma metastasis. Expression levels of TALDO1 were higher in the serum of metastatic hepatocellular carcinoma patients compared to controls [75]. Certain genetic polymorphisms in TALDO1 are associated with squamous cell carcinoma of the head and neck [76]. TALDO1 may also play a role in cancer drug resistance; higher levels of TALDO1 expression were associated with poor response to BC HER2 inhibitors in BC patients. The suppression of TALDO1 increased susceptibility to HER2 inhibition cell lines with HER2 amplification [77]. It has been proposed that TALDO1 can potentially be exploited as a biomarker or target for combination therapy in BC. We demonstrated that the specific activity of TALDO1 in cellular protein extracts and MV fractions of MDA-MB-231 was higher than its activity in MV fractions of non-cancerous MCF10A cell line. Our results support reported findings, and that TALDO1 might be a potential therapeutic target in BC.

BLMH is a cysteine aminopeptidase that was discovered for its ability to inactivate bleomycin [78,79]. Bleomycin is a small glycopeptide antibiotic used in combination therapy for the treatment of Hodgkin’s lymphoma, non-Hodgkin’s lymphoma, testicular cancer, ovarian cancer, and cervical cancer [80,81]. The antitumor effect of bleomycin is most likely caused by its ability to bind to DNA and induce the formation of toxic DNA lesions via a free radical [82,83]. Bleomycin hydrolase and poly(ADP-ribose) polymerase-1 are reported to participate in the Ubc9-mediated resistance against chemotherapy agents in human breast carcinoma MCF-7 cells [84]. Our results demonstrate a higher expression of BLMH in cancerous MDA-MB-231 MVs compared to MCF10A MVs. These results indicate that MVs may play a role in bleomycin resistance in BC.

This study and previously published work [30,85] identified the differences in the proteome between cancerous and non-cancerous breast cell line-derived EVs. We identified 87 and 112 proteins from sEVs [30] and MVs, respectively, that are related to cancer. This discovery presents a framework for future in-depth studies of each of these proteins (e.g., Table S2) from cancer-derived EVs to investigate whether they may promote cancer progression, invasion, and metastasis, remodeling of the tumor microenvironment, or angiogenesis. Such research can lead to a better understanding of the role of EVs in cancer biology. Moreover, some of these candidate proteins can be later explored for their efficacy in diagnostic or therapeutic applications for BC. The big challenge is to implement the identified candidate proteins in clinical samples by further validation and verification in large prospective studies [86]. In our present study, we found that some enzymes identified from MV fractions were already proposed to play a role in cancer therapy as therapeutic targets (OAT, TALDO1) and resistance against chemotherapy agents (BLMH). Future goals are to extensively examine and validate whether the MV enzymes could be transferred while maintaining biological activity and to investigate their biological roles in recipient cells by eventually causing physiological changes. Following this approach, EVs may serve as a source of bioactive proteins that can be used in cancer therapy, as already proposed for the treatment of several diseases [87].

## 5. Conclusions

Although EVs are known to mediate diverse biological functions, including immunomodulation, cancer progression, and epigenetic reprogramming [88], the activity of enzymes in EVs has not been thoroughly investigated. Here, we studied the enzymatic activity of OAT and TALDO1 and validated the expression level of BLMH in breast cell line-derived EVs. Both enzymes showed a significantly higher specific enzymatic activity, and BLMH exhibited higher expression in MDA-MB-231-derived MVs, suggesting they may play a role in metabolic adaptation of BC cells. Therefore, it is not surprising that these enzymes have been proposed as therapeutic targets for cancer therapy. The obtained proteomics data can be a valuable resource for the broader functional exploration of EV proteins in BC. Our findings justify further investigation of these enzymes, especially in EVs, as promising drug targets for BC treatment.

## Data Availability

The raw MS data presented in this study is openly available at the PRIDE repository. This data can be found here: https://www.ebi.ac.uk/pride/archive/projects/PXD023653.

## Supplementary Materials

The following are available online at https://www.mdpi.com/2227-9059/9/2/107/s1, Figure S1. EVs were isolated from vesicle-enriched media by differential ultracentrifugation. MVs are collected after the 15,000 g spin, while sEVs are resuspended following the 100,000 g spin. Figure S2. The Venn diagram illustrates the overlap and number of identified MV proteins derived from MDA-MB-231 and MCF10A cell lines. Figure S3. GO annotations for (A) MCF10A cellular localization, (B) MDA-MB-231 cellular localization, (C) MCF10A biological processes, and (D) MDA-MB-231 biological processes. *p*-Values were calculated using Fisher’s exact test and were obtained from DAVID functional annotation. Figure S4. Flow cytometry analysis of EV-containing cell culture supernatant. MDA-MB-231 cell culture supernatant (A) and MCF10A cell culture supernatant (B) with Copine-8 and Anti-Calreticulin antibodies. (C) and (D) Media obtained from MDA-MB-231 cells and MCF10A media, respectively, lysed with 0.1% Triton then stained with Copine-8 and Anti-Calreticulin antibodies. Figure S5. Time course of OAT and TALDO1 activity using the protein extracts from MDA-MB-231 cell-free extract CFE. Table S1. MaxQuant *proteinGroups* output file for all identified proteins from sEV and MV fractions in MCF10A and MDA-MB-231 cell lines. Contaminants and reverse proteins were put to the end. Table S2. In total, 112 proteins associated with cancer diseases were obtained from the DisGeNET human diseases database. Table S3. Only 89 proteins found in MDA-MB-231-derived MVs. Bolded proteins were validated using enzymatic assays. Table S4. A list of proteins identified as mitochondrial by their GO csellular localization terms from 89 proteins unique to MDA-MB-231 MVs. Table S5. Activities of the enzymes of the OAT and TALDO1 in the different protein extracts from cell lines and their MV and sEV fractions.
