# Supplementary material for: Breast Cancer-Derived Microvesicles Are the Source of Functional Metabolic Enzymes as Potential Targets for Cancer Therapy
Source: Biomedicines. 2021 Jan 22;9(2):107. doi: 10.3390/biomedicines9020107 (PMC7910888; doi:10.3390/biomedicines9020107)
Supplement: Supplementary file 1 [file biomedicines-09-00107-s001.zip › Supplementary materials.docx]

**Supplementary Material**

**Breast cancer-derived microvesicles are the source of functional metabolic enzymes as potential targets for cancer therapy**

Yousef Risha, Vanessa Susevski, Nico Hüttmann, Suttinee Poolsup, Zoran Minic, Maxim V. Berezovski


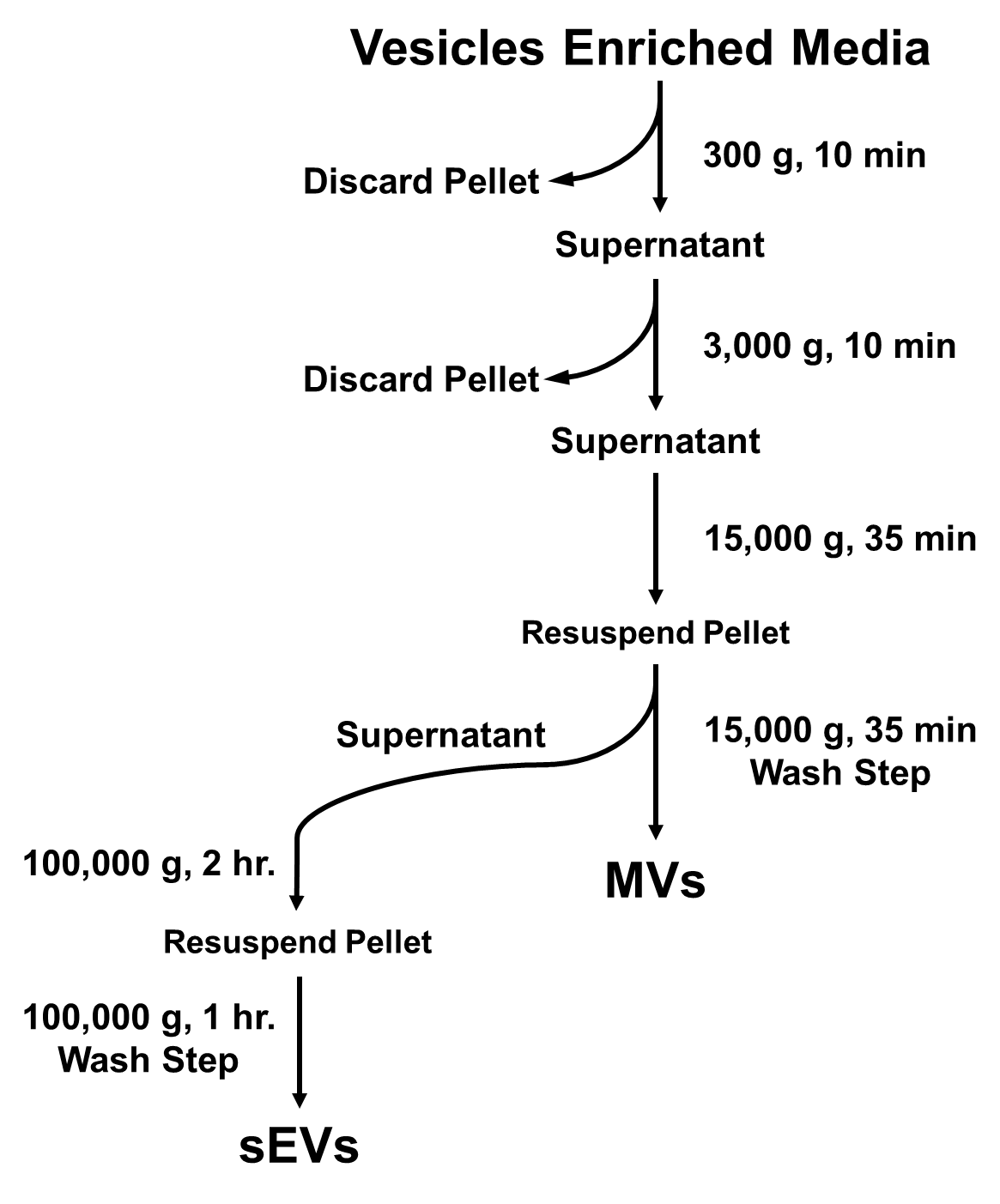


**Figure S1.** EVs were isolated from vesicle enriched media by differential ultracentrifugation. MVs are collected after the 15,000 *g* spin while sEVs are resuspended following the 100,000 *g* spin.


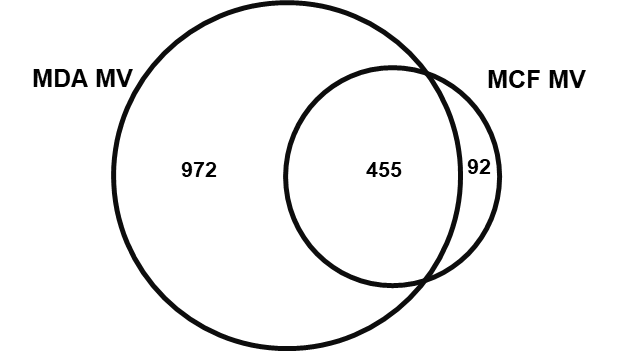


**Figure S2.** The Venn diagram illustrates the overlap and number of identified MV proteins derived from MDA-MB-231(MDA) and MCF10A (MCF) cell lines.

| **a.** | 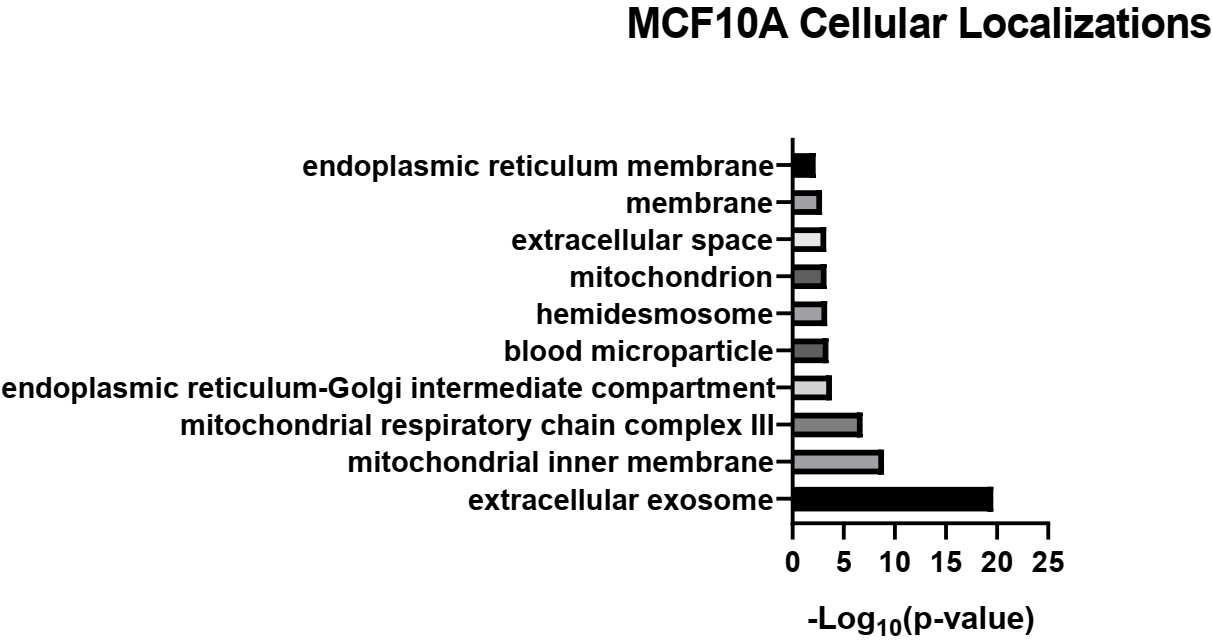 | **b.** | 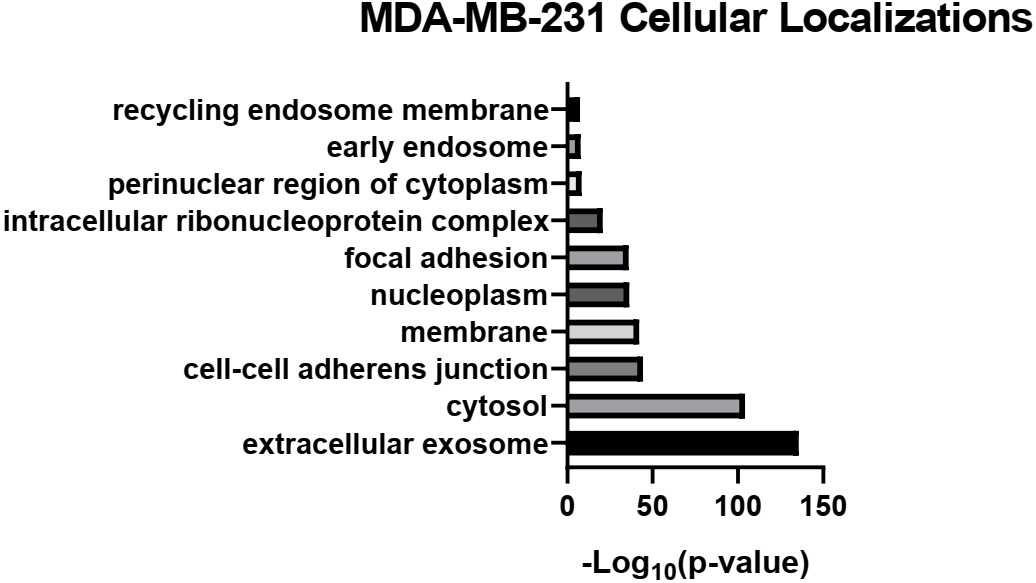 | |
| --- | --- | --- | --- | --- |
|  |  |  |  | |
| **c.** | 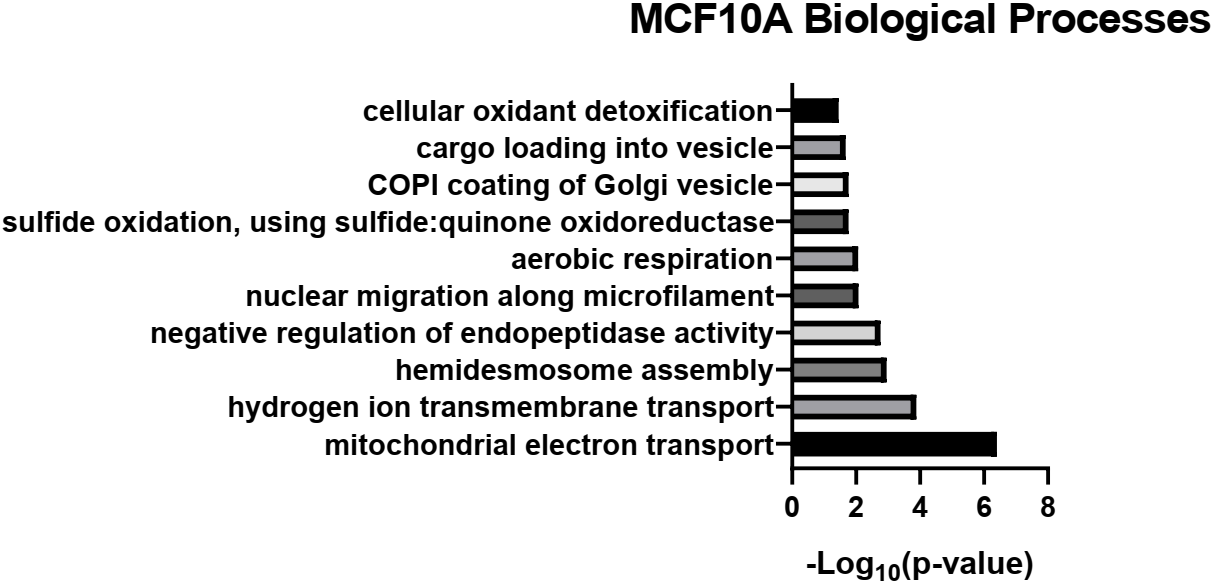 | **d.** | 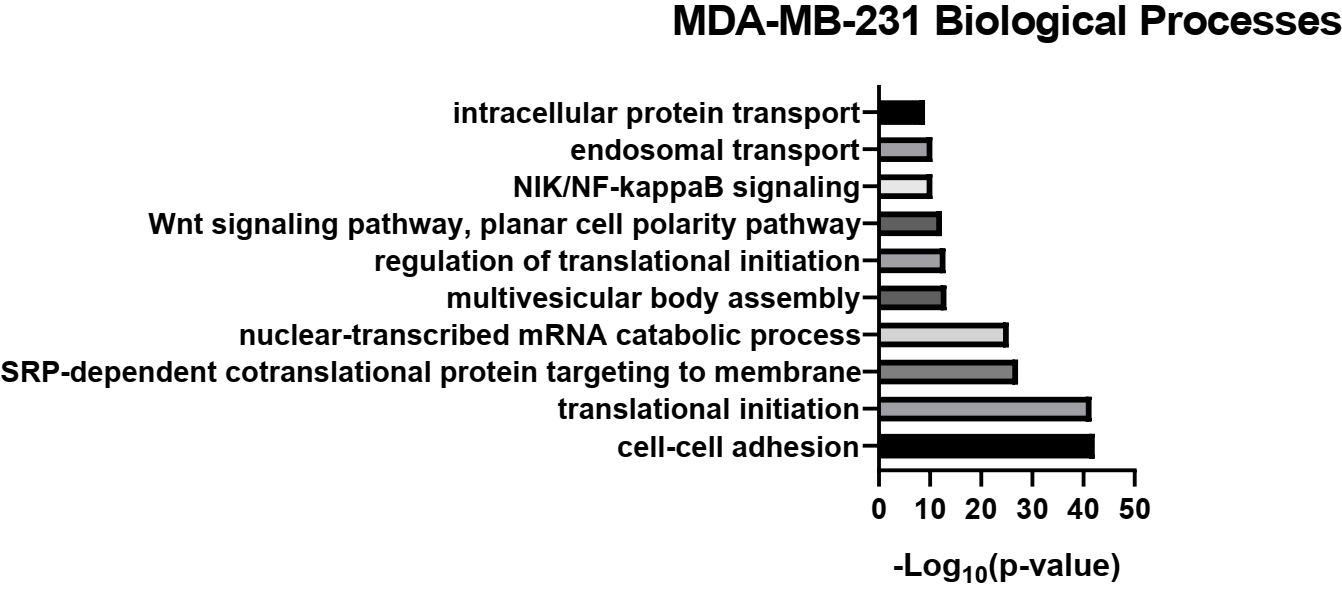 | |
| **Figure S3.** GO annotations for A. MCF10A cellular localization, B. MDA-MB-231 cellular localization, C. MCF10A biological processes, and D. MDA-MB-231 biological processes. *P*-values were calculated using Fisher’s exact test and were obtained from DAVID functional annotation. | | | |  |


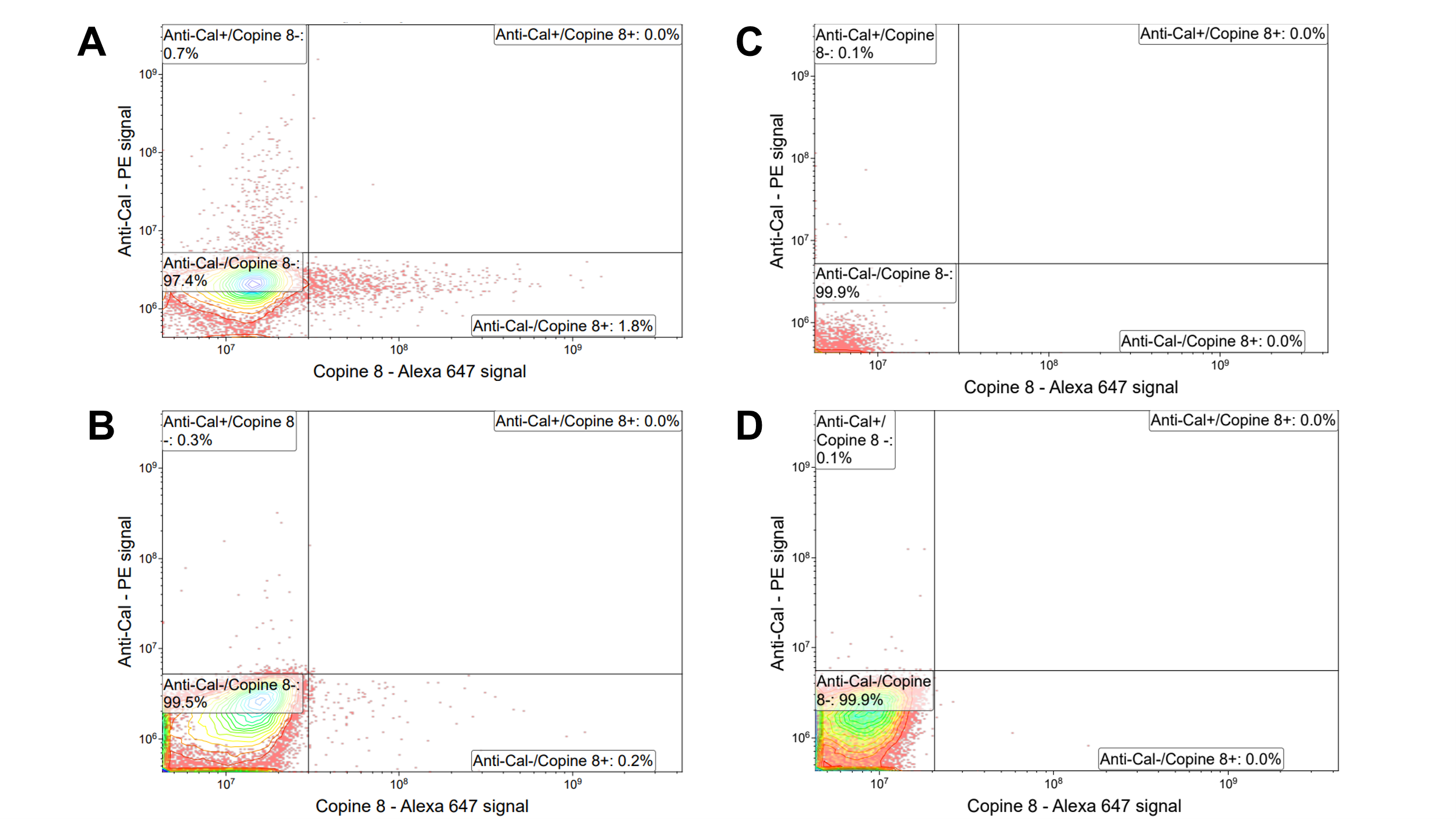


**Figure S4.** Flow cytometry analysis of EV-containing cell culture supernatant. MDA-MB-231 cell culture supernatant (A) and MCF10A cell culture supernatant (B) with Copine-8 and Anti-Calreticulin antibodies. (C) and (D) Media obtained from MDA-MB-231 cells and MCF10A media, respectively, lysed with 0.1% Triton then stained with Copine-8 and Anti-Calreticulin antibodies.

**
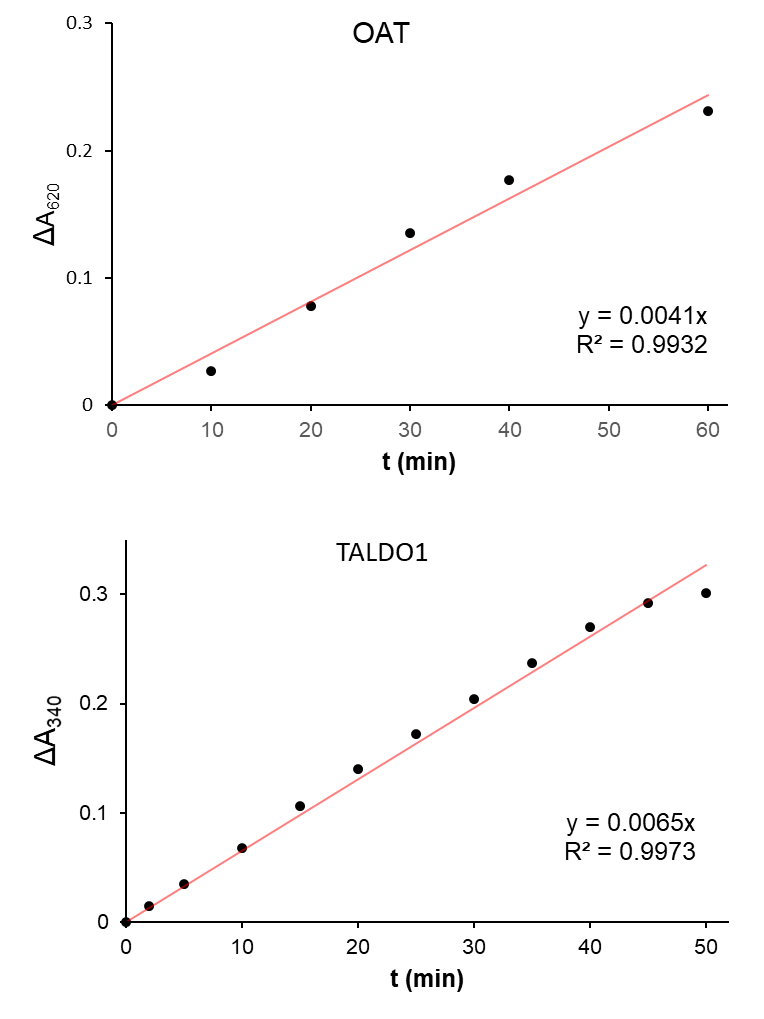
**

**Figure S5.** Time course of OAT and TALDO1 activity using protein extracts from MDA-MB-231 CFE.

|  |  |  |
| --- | --- | --- |
| Table S2. 112 proteins associated with cancer diseases were obtained from the DisGeNET human diseases database. | | |
| Protein | **Gene Symbol** | **Protein Description** |
| P21399 | ACO1 | Cytoplasmic aconitate hydratase |
| O14672 | ADAM10 | Disintegrin and metalloproteinase domain-containing protein 10 |
| Q9UHI8 | ADAMTS1 | A disintegrin and metalloproteinase with thrombospondin motifs 1 |
| Q9BV57 | ADI1 | 1,2-dihydroxy-3-keto-5-methylthiopentene dioxygenase |
| P23526 | AHCY | Adenosylhomocysteinase |
| P00568 | AK1 | Adenylate kinase isoenzyme 1 |
| Q02952 | AKAP12 | A-kinase anchor protein 12 |
| Q4VCS5 | AMOT | Angiomotin |
| P02649 | APOE | Apolipoprotein E |
| Q96GD4 | AURKB | Aurora kinase B |
| Q9NWV8 | BABAM1 | BRISC and BRCA1-A complex member 1 |
| Q07812 | BAX | Apoptosis regulator BAX |
| P54289 | CACNA2D1 | Voltage-dependent calcium channel subunit alpha-2/delta-1;Voltage-dependent calcium channel subunit alpha-2-1;Voltage-dependent calcium channel subunit delta-1 |
| P27708 | CAD | CAD protein;Glutamine-dependent carbamoyl-phosphate synthase;Aspartate carbamoyltransferase;Dihydroorotase |
| Q9Y5S2 | CDC42BPB | Serine/threonine-protein kinase MRCK beta |
| Q9NZZ3 | CHMP5 | Charged multivesicular body protein 5 |
| P12277 | CKB | Creatine kinase B-type |
| P55060 | CSE1L | Exportin-2 |
| P68400 | CSNK2A1 | Casein kinase II subunit alpha |
| P19784 | CSNK2A2 | Casein kinase II subunit alpha |
| P35222 | CTNNB1 | Catenin beta-1 |
| Q92796 | DLG3 | Disks large homolog 3 |
| P11532 | DMD | Dystrophin |
| P50570 | DNM2 | Dynamin-2 |
| P47813 | EIF1AX | Eukaryotic translation initiation factor 1A, X-chromosomal |
| P60228 | EIF3E | Eukaryotic translation initiation factor 3 subunit E |
| Q14240 | EIF4A2 | Eukaryotic initiation factor 4A-II;Eukaryotic initiation factor 4A-II, N-terminally processed |
| Q04637 | EIF4G1 | Eukaryotic translation initiation factor 4 gamma 1 |
| P55010 | EIF5 | Eukaryotic translation initiation factor 5 |
| P63241 | EIF5A | Eukaryotic translation initiation factor 5A-1 |
| Q8N8S7 | ENAH | Protein enabled homolog |
| Q96RT1 | ERBIN | Protein LAP2 |
| P39748 | FEN1 | Flap endonuclease 1 |
| P35637 | FUS | RNA-binding protein FUS |
| P06241 | FYN | Tyrosine-protein kinase Fyn |
| P11413 | G6PD | Glucose-6-phosphate 1-dehydrogenase |
| P17302 | GJA1 | Gap junction alpha-1 protein |
| Q14344 | GNA13 | Guanine nucleotide-binding protein subunit alpha-13 |
| P50148 | GNAQ | Guanine nucleotide-binding protein G(q) subunit alpha |
| Q96EK6 | GNPNAT1 | Glucosamine 6-phosphate N-acetyltransferase |
| Q8NBJ4 | GOLM1 | Golgi membrane protein 1 |
| P49841 | GSK3B | Glycogen synthase kinase-3 beta |
| P78417 | GSTO1 | Glutathione S-transferase omega-1 |
| P07305 | H1-0 | Histone H1.0;Histone H1.0, N-terminally processed |
| P16403 | H1-2 | Histone H1.2 |
| P12081 | HARS1 | Histidine--tRNA ligase, cytoplasmic |
| O75874 | IDH1 | Isocitrate dehydrogenase [NADP] cytoplasmic |
| P11717 | IGF2R | Cation-independent mannose-6-phosphate receptor |
| Q13418 | ILK | Integrin-linked protein kinase |
| P12268 | IMPDH2 | Inosine-5-monophosphate dehydrogenase 2 |
| Q9NQS7 | INCENP | Inner centromere protein |
| O00410 | IPO5 | Importin-5 |
| Q13683 | ITGA7 | Integrin alpha-7;Integrin alpha-7 heavy chain;Integrin alpha-7 light chain;Integrin alpha-7 70 kDa form |
| P33176 | KIF5B | Kinesin-1 heavy chain |
| P52292 | KPNA2 | Importin subunit alpha-1 |
| P55268 | LAMB2 | Laminin subunit beta-2 |
| P11047 | LAMC1 | Laminin subunit gamma-1 |
| Q96AG4 | LRRC59 | Leucine-rich repeat-containing protein 59 |
| Q32MZ4 | LRRFIP1 | Leucine-rich repeat flightless-interacting protein 1 |
| Q7KZI7 | MARK2 | Serine/threonine-protein kinase MARK2 |
| O95297 | MPZL1 | Myelin protein zero-like protein 1 |
| Q7Z406 | MYH14 | Myosin-14 |
| P04181 | OAT | Ornithine aminotransferase, mitochondrial;Ornithine aminotransferase, hepatic form;Ornithine aminotransferase, renal form |
| Q16625 | OCLN | Occludin |
| Q9NTK5 | OLA1 | Obg-like ATPase 1 |
| Q9NWQ8 | PAG1 | Phosphoprotein associated with glycosphingolipid-enriched microdomains 1 |
| Q96IZ0 | PAWR | PRKC apoptosis WT1 regulator protein |
| P12004 | PCNA | Proliferating cell nuclear antigen |
| P36871 | PGM1 | Phosphoglucomutase-1 |
| Q13492 | PICALM | Phosphatidylinositol-binding clathrin assembly protein |
| O00592 | PODXL | Podocalyxin |
| Q15181 | PPA1 | Inorganic pyrophosphatase |
| P49593 | PPM1F | Protein phosphatase 1F |
| Q9Y617 | PSAT1 | Phosphoserine aminotransferase |
| P49810 | PSEN2 | Presenilin-2;Presenilin-2 NTF subunit;Presenilin-2 CTF subunit |
| O75116 | ROCK2 | Rho-associated protein kinase 2 |
| P50914 | RPL14 | 60S ribosomal protein L14 |
| Q9Y3A5 | SBDS | Ribosome maturation protein SBDS |
| P31431 | SDC4 | Syndecan-4 |
| P31040 | SDHA | Succinate dehydrogenase [ubiquinone] flavoprotein subunit, mitochondrial |
| Q01105 | SET | Protein SET |
| P34897 | SHMT2 | Serine hydroxymethyltransferase, mitochondrial |
| P55011 | SLC12A2 | Solute carrier family 12 member 2 |
| P41440 | SLC19A1 | Folate transporter 1 |
| Q99808 | SLC29A1 | Equilibrative nucleoside transporter 1 |
| P11166 | SLC2A1 | Solute carrier family 2, facilitated glucose transporter member 1 |
| P04920 | SLC4A2 | Anion exchange protein 2 |
| P30825 | SLC7A1 | High affinity cationic amino acid transporter 1 |
| Q9H2G2 | SLK | STE20-like serine/threonine-protein kinase |
| O60264 | SMARCA5 | SWI/SNF-related matrix-associated actin-dependent regulator of chromatin subfamily A member 5 |
| Q9UQE7 | SMC3 | Structural maintenance of chromosomes protein 3 |
| Q9Y5X3 | SNX5 | Sorting nexin-5 |
| P12931 | SRC | Proto-oncogene tyrosine-protein kinase Src |
| Q07955 | SRSF1 | Serine/arginine-rich splicing factor 1 |
| Q13247 | SRSF6 | Serine/arginine-rich splicing factor 6 |
| Q9P289 | STK26 | Serine/threonine-protein kinase 26 |
| P16949 | STMN1 | Stathmin |
| P63165 | SUMO1 | Small ubiquitin-related modifier 1 |
| Q99426 | TBCB | Tubulin-folding cofactor B |
| P17987 | TCP1 | T-complex protein 1 subunit alpha |
| P02786 | TFRC | Transferrin receptor protein 1;Transferrin receptor protein 1, serum form |
| P11387 | TOP1 | DNA topoisomerase 1 |
| P11388 | TOP2A | DNA topoisomerase 2-alpha |
| Q02880 | TOP2B | DNA topoisomerase 2-beta |
| Q13641 | TPBG | Trophoblast glycoprotein |
| P0DN76 | U2AF1 | Splicing factor U2AF 35 kDa subunit-like protein |
| P09936 | UCHL1 | Ubiquitin carboxyl-terminal hydrolase isozyme L1 |
| P15374 | UCHL3 | Ubiquitin carboxyl-terminal hydrolase isozyme L3 |
| O60763 | USO1 | General vesicular transport factor p115 |
| P13611 | VCAN | Versican core protein |
| Q9BQA1 | WDR77 | Methylosome protein 50 |
| P12956 | XRCC6 | X-ray repair cross-complementing protein 6 |

|  |  |  |
| --- | --- | --- |
| Table S3. 89 proteins only found in MDA-MB-231 derived MVs. Bolded proteins were validated using enzymatic assays. | | |
| Protein | **Gene Symbol** | **Protein Description** |
| Q01105 | SET | Protein SET;Protein SETSIP |
| P04181 | **OAT** | **Ornithine aminotransferase, mitochondrial;Ornithine aminotransferase, hepatic form;Ornithine aminotransferase, renal form** |
| P34897 | SHMT2 | Serine hydroxymethyltransferase, mitochondrial |
| Q13867 | **BLMH** | **Bleomycin hydrolase** |
| Q8IY81 | FTSJ3 | pre-rRNA processing protein FTSJ3 |
| P37837 | **TALDO1** | **Transaldolase** |
| O43504 | LAMTOR5 | Ragulator complex protein LAMTOR5 |
| Q04637 | EIF4G1 | Eukaryotic translation initiation factor 4 gamma 1 |
| Q13765 | NACA | Nascent polypeptide-associated complex subunit alpha;Nascent polypeptide-associated complex subunit alpha, muscle-specific form |
| Q9BZE4 | GTPBP4 | Nucleolar GTP-binding protein 1 |
| Q9H0A0 | NAT10 | N-acetyltransferase 10 |
| P16278 | GLB1 | Beta-galactosidase |
| Q96IZ0 | PAWR | PRKC apoptosis WT1 regulator protein |
| Q9H1E3 | NUCKS1 | Nuclear ubiquitous casein and cyclin-dependent kinase substrate 1 |
| Q9Y547 | HSPB11 | Intraflagellar transport protein 25 homolog |
| Q9UNZ2 | NSFL1C | NSFL1 cofactor p47 |
| O15069 | NACAD | NAC-alpha domain-containing protein 1 |
| O00629 | KPNA4 | Importin subunit alpha-3 |
| Q99439 | CNN2 | Calponin-2 |
| P63165 | SUMO1 | Small ubiquitin-related modifier 1 |
| O43396 | TXNL1 | Thioredoxin-like protein 1 |
| Q9UHB6 | LIMA1 | LIM domain and actin-binding protein 1 |
| Q14C86 | GAPVD1 | GTPase-activating protein and VPS9 domain-containing protein 1 |
| Q96C36 | PYCR2 | Pyrroline-5-carboxylate reductase 2 |
| Q9H0W5 | CCDC8 | Coiled-coil domain-containing protein 8 |
| Q9NWV8 | BABAM1 | BRISC and BRCA1-A complex member 1 |
| P46108 | CRK | Adapter molecule crk |
| Q8IYS1 | PM20D2 | Peptidase M20 domain-containing protein 2 |
| P22612 | PRKACG | cAMP-dependent protein kinase catalytic subunit gamma |
| P13804 | ETFA | Electron transfer flavoprotein subunit alpha, mitochondrial |
| P62191 | PSMC1 | 26S protease regulatory subunit 4 |
| Q9Y262 | EIF3L | Eukaryotic translation initiation factor 3 subunit L |
| O43663 | PRC1 | Protein regulator of cytokinesis 1 |
| P52597 | HNRNPF | Heterogeneous nuclear ribonucleoprotein F;Heterogeneous nuclear ribonucleoprotein F, N-terminally processed |
| P15170 | GSPT1 | Eukaryotic peptide chain release factor GTP-binding subunit ERF3A;Eukaryotic peptide chain release factor GTP-binding subunit ERF3B |
| Q12931 | TRAP1 | Heat shock protein 75 kDa, mitochondrial |
| Q8N912 | NRAC | Nutritionally-regulated adipose and cardiac enriched protein homolog |
| Q93050 | ATP6V0A1 | V-type proton ATPase 116 kDa subunit a isoform 1 |
| P43034 | PAFAH1B1 | Platelet-activating factor acetylhydrolase IB subunit alpha |
| Q8IYT4 | KATNAL2 | Katanin p60 ATPase-containing subunit A-like 2 |
| P48147 | PREP | Prolyl endopeptidase |
| Q6IBS0 | TWF2 | Twinfilin-2 |
| P55809 | OXCT1 | Succinyl-CoA:3-ketoacid coenzyme A transferase 1, mitochondrial |
| Q12905 | ILF2 | Interleukin enhancer-binding factor 2 |
| P24666 | ACP1 | Low molecular weight phosphotyrosine protein phosphatase |
| Q9NS69 | TOMM22 | Mitochondrial import receptor subunit TOM22 homolog |
| Q9UHA4 | LAMTOR3 | Ragulator complex protein LAMTOR3 |
| O00303 | EIF3F | Eukaryotic translation initiation factor 3 subunit F |
| Q3SXY8 | ARL13B | ADP-ribosylation factor-like protein 13B |
| P21399 | ACO1 | Cytoplasmic aconitate hydratase |
| Q13907 | IDI1 | Isopentenyl-diphosphate Delta-isomerase 1 |
| P21266 | GSTM3 | Glutathione S-transferase Mu 3 |
| Q9BW83 | IFT27 | Intraflagellar transport protein 27 homolog |
| P31040 | SDHA | Succinate dehydrogenase [ubiquinone] flavoprotein subunit, mitochondrial |
| O75844 | ZMPSTE24 | CAAX prenyl protease 1 homolog |
| O15392 | BIRC5 | Baculoviral IAP repeat-containing protein 5 |
| P00568 | AK1 | Adenylate kinase isoenzyme 1 |
| Q9NSD9 | FARSB | Phenylalanine--tRNA ligase beta subunit |
| P28161 | GSTM2 | Glutathione S-transferase Mu 2 |
| P31942 | HNRNPH3 | Heterogeneous nuclear ribonucleoprotein H3 |
| Q96EK6 | GNPNAT1 | Glucosamine 6-phosphate N-acetyltransferase |
| Q01581 | HMGCS1 | Hydroxymethylglutaryl-CoA synthase, cytoplasmic |
| Q9GZT8 | NIF3L1 | NIF3-like protein 1 |
| P06132 | UROD | Uroporphyrinogen decarboxylase |
| Q12906 | ILF3 | Interleukin enhancer-binding factor 3 |
| Q9GZZ9 | UBA5 | Ubiquitin-like modifier-activating enzyme 5 |
| Q9UJS0 | SLC25A13 | Calcium-binding mitochondrial carrier protein Aralar2 |
| O75534 | CSDE1 | Cold shock domain-containing protein E1 |
| Q9UNS2 | COPS3 | COP9 signalosome complex subunit 3 |
| Q9NRV9 | HEBP1 | Heme-binding protein 1 |
| Q9NS86 | LANCL2 | LanC-like protein 2 |
| Q68EM7 | ARHGAP17 | Rho GTPase-activating protein 17 |
| O43760 | SYNGR2 | Synaptogyrin-2 |
| P14550 | AKR1A1 | Alcohol dehydrogenase [NADP(+)] |
| P49354 | FNTA | Protein farnesyltransferase/geranylgeranyltransferase type-1 subunit alpha |
| P51452 | DUSP3 | Dual specificity protein phosphatase 3 |
| Q08257 | CRYZ | Quinone oxidoreductase |
| Q9BX68 | HINT2 | Histidine triad nucleotide-binding protein 2, mitochondrial |
| Q9UHY7 | ENOPH1 | Enolase-phosphatase E1 |
| O43847 | NRD1 | Nardilysin |
| Q8NI36 | WDR36 | WD repeat-containing protein 36 |
| Q9Y2G3 | ATP11B | Probable phospholipid-transporting ATPase IF |
| Q9H7C9 | AAMDC | Mth938 domain-containing protein |
| O60343 | TBC1D4 | TBC1 domain family member 4 |
| P27816 | MAP4 | Microtubule-associated protein 4 |
| Q13206 | DDX10 | Probable ATP-dependent RNA helicase DDX10 |
| Q96DA6 | DNAJC19 | Mitochondrial import inner membrane translocase subunit TIM14 |
| Q96EY1 | DNAJA3 | DnaJ homolog subfamily A member 3, mitochondrial |
| Q9BVM2 | DPCD | Protein DPCD |

|  |  |  |
| --- | --- | --- |
| Table S4. A list of proteins identified as mitochondrial by their GO cellular localization terms from 89 proteins unique to MDA-MB-231 MVs. | | |
| Protein | **Gene Symbol** | **Protein Description** |
| P55809 | OXCT1 | 3-oxoacid CoA-transferase 1 |
| Q96EY1 | DNAJA3 | DnaJ heat shock protein family (Hsp40) member A3 |
| Q96DA6 | DNAJC19 | DnaJ heat shock protein family (Hsp40) member C19 |
| Q9GZT8 | NIF3L1 | NGG1 interacting factor 3 like 1 |
| Q12931 | TRAP1 | TNF receptor associated protein 1 |
| P21399 | ACO1 | aconitase 1 |
| P13804 | ETFA | electron transfer flavoprotein alpha subunit |
| Q9BX68 | HINT2 | histidine triad nucleotide binding protein 2 |
| Q12906 | ILF3 | interleukin enhancer binding factor 3 |
| O43847 | NRDC | nardilysin convertase |
| P04181 | OAT | ornithine aminotransferase |
| Q96C36 | PYCR2 | pyrroline-5-carboxylate reductase family member 2 |
| P34897 | SHMT2 | serine hydroxymethyltransferase 2 |
| Q9UJS0 | SLC25A13 | solute carrier family 25 member 13 |
| P31040 | SDHA | succinate dehydrogenase complex flavoprotein subunit A |
| O43396 | TXNL1 | thioredoxin like 1 |
| Q9NS69 | TOMM22 | translocase of outer mitochondrial membrane 22 |

**Table S5**. Activities of OAT and TALDO1 in the different protein extracts from cell and their derived MV and sEV fractions. The numbers in parentheses indicate the number of biological replicates. ND, not detected.

| Sample types | Specific activities (nmol/min/mg) | |
| --- | --- | --- |
|  | OAT | TALDO1 |
| MCF10A CFE | 1.290 ± 0.245 (4) | 1.519 ± 1.006 (4) |
| MCF10A MV | 0.597 ± 0.733 (4) | 1.382 ± 0.211 (4) |
| MCF10A sEV | ND | 0.092 ± 0.025 (4) |
|  |  |  |
| MDA-MB-231 CFE | 2.842 ± 0.674 (4) | 10.763 ± 4.554 (4) |
| MDA-MB-231 MV | 3.74 ± 1.129 (4) | 4.626 ± 1.586 (4) |
| MDA-MB-231 sEV | ND | 0.371 ± 0.126 (4) |
